# Genome-wide identification of candidate copy number polymorphism genes associated with complex traits of Tibetan-sheep

**DOI:** 10.1038/s41598-023-44402-1

**Published:** 2023-10-12

**Authors:** Dehong Tian, De Sun, Qianben Ren, Pei Zhang, Zian Zhang, Wenkui Zhang, Haizhou Luo, Xue Li, Buying Han, Dehui Liu, Kai Zhao

**Affiliations:** 1 Qinghai Provincial Key Laboratory of Animal Ecological Genomics, Key Laboratory of Adaptation and Evolution of Plateau Biota, Northwest Institute of Plateau Biology, Chinese Academy of Sciences, Xining, 810001 Qinghai China; 2 University of Chinese Academy of Sciences, Beijing, 100049 China; 3 Animal Husbandry and Veterinary Station of Huzhu County of Qinghai Province, Huzhu, 810500 Qinghai China; 4 Qinghai Sheep Breeding and Promotion Service Center, Gangcha, 812300 Qinghai China; 5 Qinghai Animal and Plant Quarantine Station, Xining, 810000 Qinghai China

**Keywords:** Animal breeding, Genomics, Bioinformatics

## Abstract

Copy number variation (CNV) is a genetic structural polymorphism important for phenotypic diversity and important economic traits of livestock breeds, and it plays an important role in the desired genetic variation. This study used whole genome sequencing to detect the CNV variation in the genome of 6 local Tibetan sheep groups. We detected 69,166 CNV events and 7230 copy number variable regions (CNVRs) after merging the overlapping CNVs, accounting for 2.72% of the reference genome. The CNVR length detected ranged from 1.1 to 1693.5 Kb, with a total length of 118.69 Mb and an average length of 16.42 Kb per CNVR. Functional GO cluster analysis showed that the CNVR genes were mainly involved in sensory perception systems, response to stimulus, and signal transduction. Through CNVR-based Vst analysis, we found that the *CACNA2D3* and *CTBP1* genes related to hypoxia adaptation, the *HTR1A* gene related to coat color, and the *TRNAS-GGA* and *PIK3C3* genes related to body weight were all strongly selected. The findings of our study will contribute novel insights into the genetic structural variation underlying hypoxia adaptation and economically important traits in Tibetan sheep.

## Introduction

Tibetan sheep have formed some breeds with rich and unique morphological features (e.g., horn morphology, ear size, tail length, coat color, etc.), disease resistance, reproductive performance, and environmental adaptability in the process of adapting to the local environment by the different needs and preferences of human beings. Most important traits are complex, i.e., traits that are affected by many genes and the environment^1^. Copy number variation (CNV) is one of the sources of genetic diversity and can affect phenotypes by inducing gene structure and expression^2^. CNV refers to a structural variation type in the genome, including deletions, insertions, duplications, large-scale copy number variants, inversions, and translocations of ∼ 1 kilobase (kb) or larger^3,4^. Structural variants can impinge on functional elements in many ways, both inside and beyond the genomic interval they occur in, with significant effects on various phenotypes^5^. CNV loci encompassing genes may affect gene expression and reshape gene structure, subsequently shaping significant phenotypic variation^6,7^. Most CNVs are often recessive in the heterozygous state, and in many cases, dosage-sensitive CNVs that are upregulated and downregulated coincide with copy-number increases and decreases^8^.

Researchers have identified a large number of CNVs in different breeding livestock populations. CNVs have made an important contribution to the genetic variation in the genome, which can cause substantial phenotypic changes through multiple effects, such as gene dosage modification, gene structure disturbance, gene fusion, gene disruption, and positional effects^9,10^. Genome-wide analysis of Meishan and Duroc pigs identified 17.17 Mb of 6387 CNVRs that only existed in Meishan pigs, which overlapped with reproductively related genes encoding the aryl hydrocarbon receptor (*AHR*) gene. *AHR* is a candidate gene associated with reproductive traits that can be used as a genetic marker in pig breeding programs^11^. The two most significant CNVRs located on chromosome 19 were associated with milk production traits in Valle del Belice sheep^12^. A total of 222 genes were annotated within the significantly associated CNVRs, most of which played important roles in biological processes related to milk production and health-related traits^12^. One CNVR overlapping with the homeobox transcription factor *DLX3* and previously shown to be associated with curly hair in sheep was identified as the candidate CNV for the special curly fleece phenotype in Tan sheep^13^. Additionally, another experiment confirmed that CNVR22 had significantly negative effects on both *PLA2G2D* gene expression and cattle body measurements, while CNVR310 showed a significant negative association with heart girth^14^. However, little is known about further elucidating of the relationship between CNVs and production and phenotypic traits in different Chinese sheep breeds by whole-genome sequencing. In addition, there are no reports on further elucidation of CNVs in different Chinese indigenous Tibetan sheep breeds by whole genome sequencing.

**Figure 1:**
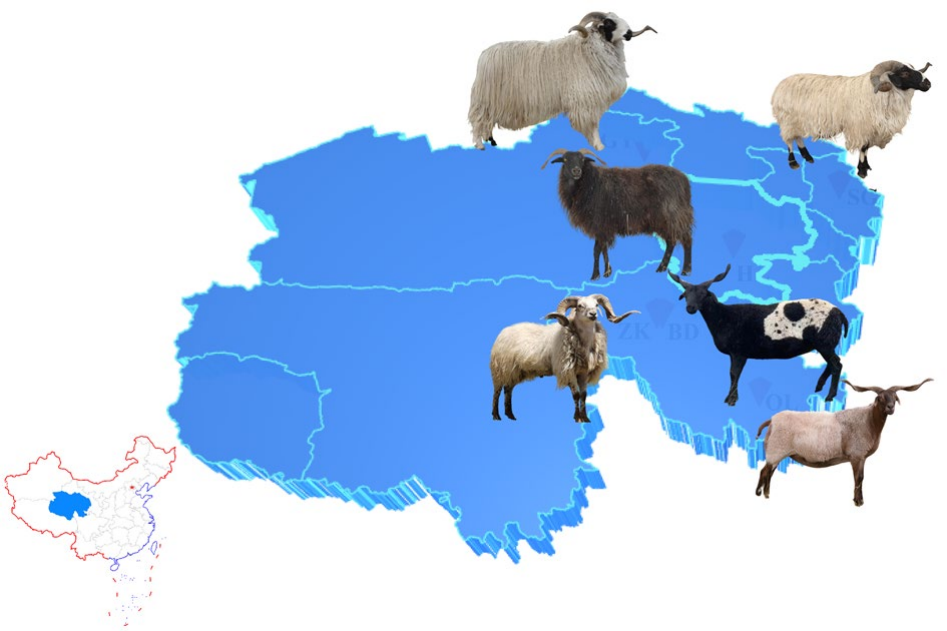
Geographic distribution of six Tibetan sheep breeds in Qinghai. The map was generated using Adobe Illustrator software.

Therefore, in this study, the CNVs of six Tibetan sheep breeds were identified by whole genome sequencing, and GWAS analyzed their production and phenotypic traits based on CNVs. In addition, genome-wide characterization of CNV analysis was conducted to establish the relationship between genomic variation and phenotype and to search for pathways or genes associated with regulating of economic traits. A significant number of overlapping CNV fragments and candidate CNVR were obtained, providing a scientific foundation for determining the formation of crucial traits in Tibetan sheep. This finding holds immense importance for safeguarding valuable germplasm resources of superior breeds and facilitating targeted breeding programs for exceptional traits (Fig. 1).

## Results

### Sequencing results and CNV identification

The raw data of the 28 Tibetan sheep samples were 2375.97 Gb, and the filtered clean data were 2254.50 Gb. The average coverage depth of the reference genome ranged from 16.75× to 23.08×. This indicates that the sequencing depth was sufficient to detect CNVs (Supplementary Table S2).

**Figure 2:**
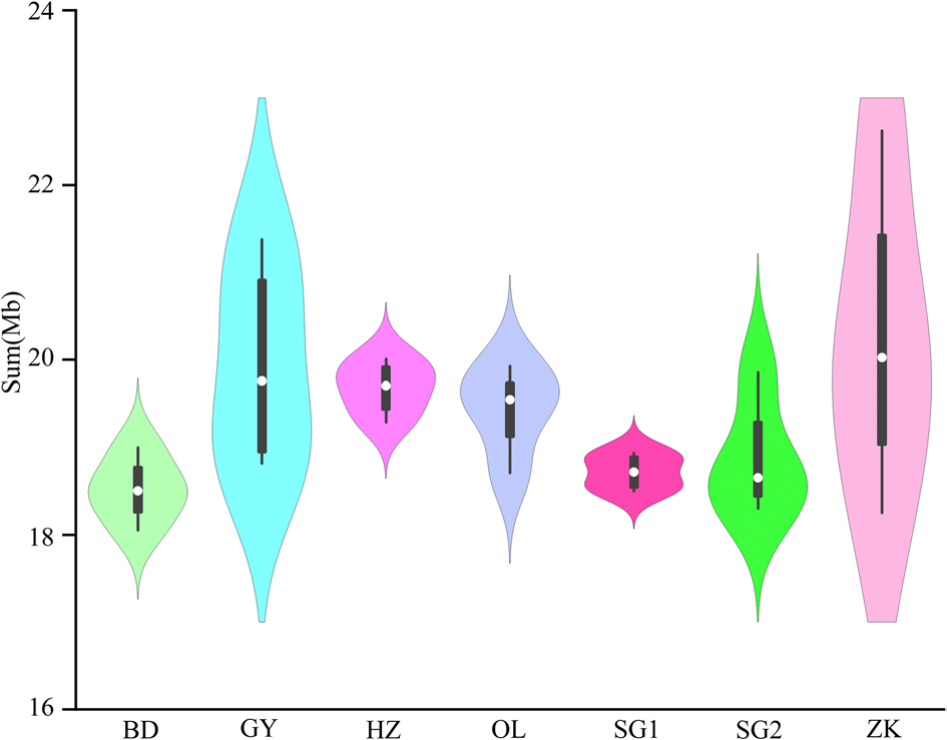
Violin plots showing distribution of the total CNV length in different group.

A total of 69,166 CNV events were detected in the 28 Tibetan sheep, with each sheep’s genome possessing 2470.71 CNVs, on average, which consisted of 20,732 duplication events and 48,434 deletion events (Table 1, Supplementary Table S3). In addition, the violin plot showed a slight difference in the distribution of CNV length among different populations. However, the total number of CNVs in the GY and ZK groups varied greatly within this population (Fig. 2).

**Table 1:** Summary of CNVs and CNVRs identified.

	Breeds	Count	Duplication	Deletion	Both	Length (Mb)	Average (kb)	Percentage of chromosome by CNVRs (%)
CNVs	BD	10,241	2775	7466	–	74.06	7.23	–
	GY	10,048	2908	7140	–	79.71	7.93	–
	HZ	9687	3015	6672	–	78.71	8.13	–
	OL	9725	2770	6955	–	77.73	7.99	–
	SG1	9933	2796	7137	–	74.87	7.54	–
	SG2	9301	2905	6396	–	75.46	8.11	–
	ZK	10,231	3563	6668	–	80.92	7.91	–
CNVRs	BD	3923	736	3114	73	26.75	6.82	1.02
	GY	4103	867	3141	95	32.45	7.91	1.24
	HZ	3912	832	2994	86	28.87	7.38	1.11
	OL	3928	772	3063	93	29.50	7.51	1.13
	SG1	4014	820	3122	72	27.83	6.93	1.07
	SG2	3641	805	2756	80	27.14	7.45	1.04
	ZK	4080	1037	2942	101	32.76	8.03	1.26

**Figure 3:**
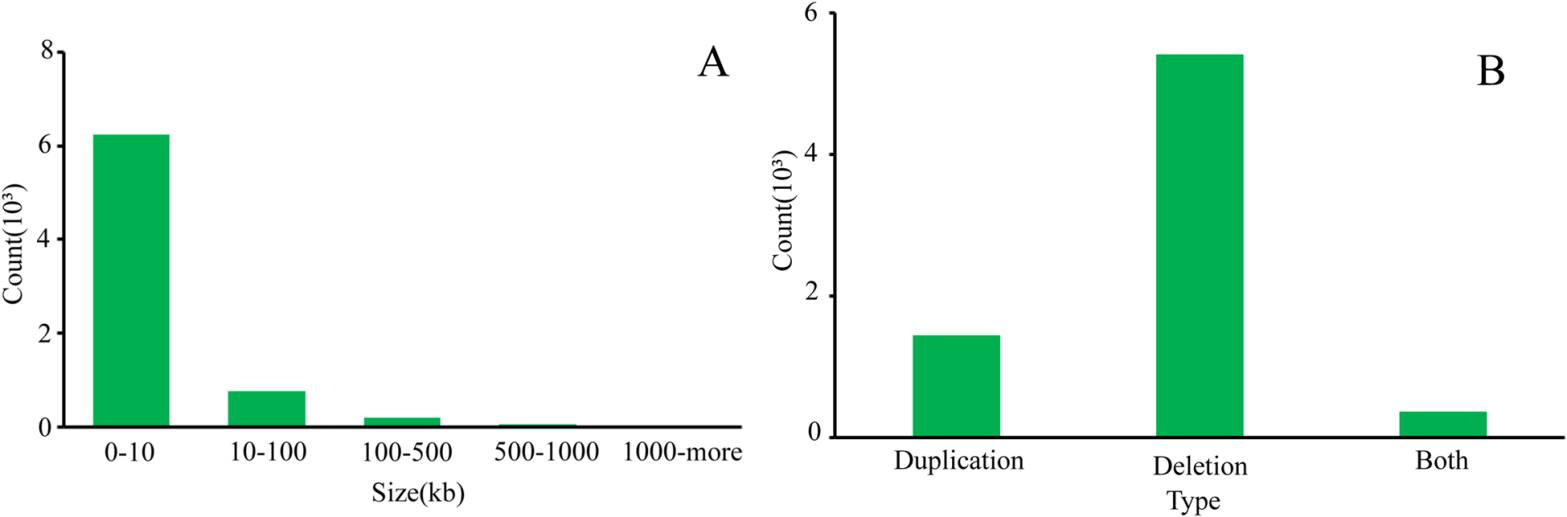
Size and type distribution of CNVRs in Tibetan sheep. (**A**): The length and frequency distribution of differential CNVRs. (**B**): The different types of CNVRs summary statistics.

**Figure 4:**
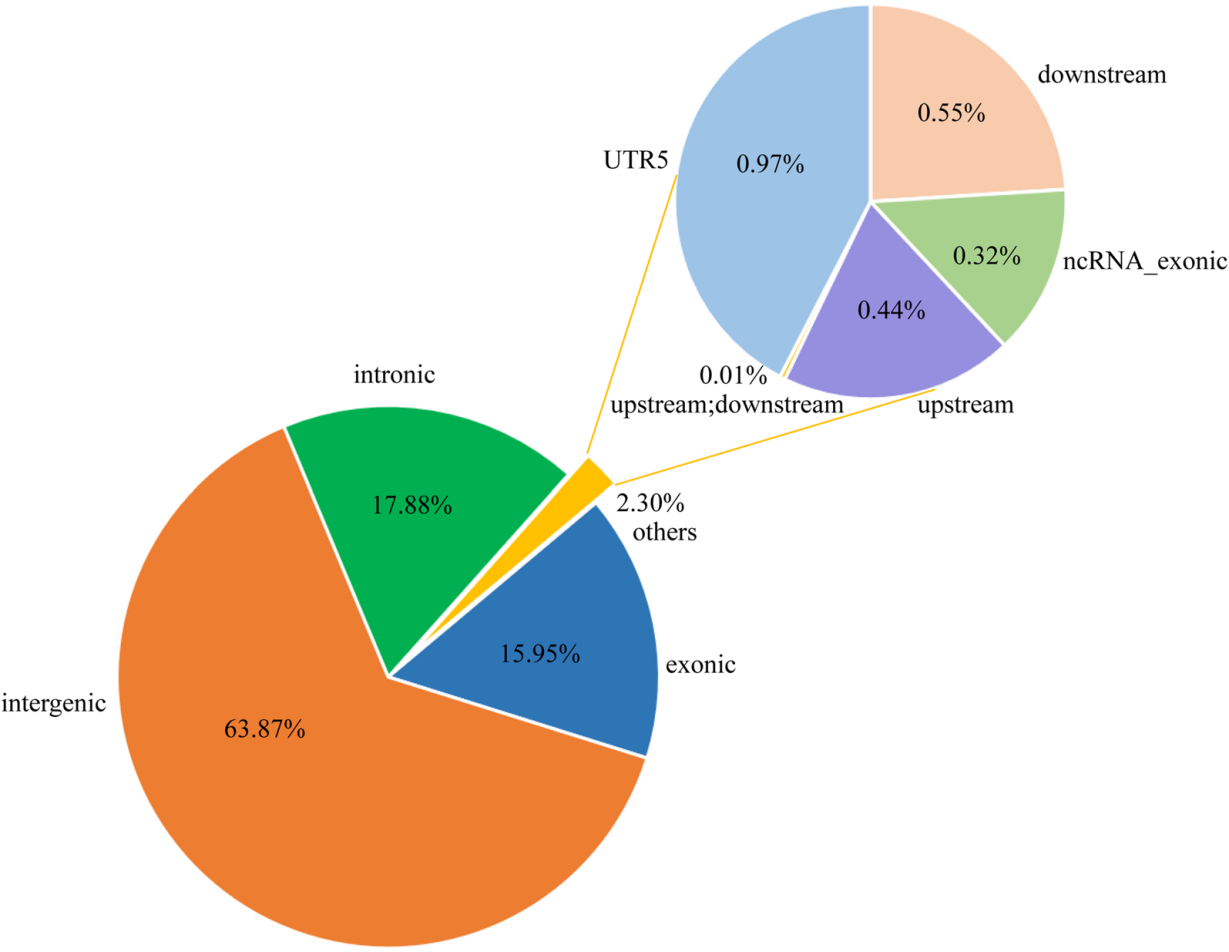
The proportion of CNVRs in different functional regions of chromosome.

After we merged the overlapping CNVs, a total of 7230 CNVRs were found; among these, 5416 CNVRs were deletions, 1450 CNVRs were duplications, and 364 CNVRs were both (Table 2, Supplementary Table S4). The size of the identified CNVRs ranged from 1.1 kb to 1693.5 kb in length. The distribution showed that 86.14% of the CNVRs ranged from 0 to 10 kb in size, 10.48% were within 10–100 kb, and 3.37% were more significant than 100 kb in length (Fig. 3A and B). In addition, 63.87% of the CNVRs were in the intergenic region, 17.88% of the variants were in the intronic region, 15.95% were in the exon region, and relatively few were in the upstream and downstream 1.9 kb regions (Fig. 4).

**Table 2:** Summary statistics and size distribution information of CNVRs.

CNVR summary statistics	Total	Duplication	Deletion	Both
Number of CNVRs	7230 (100%)	1450 (20.06%)	5416 (74.91%)	364 (5.03%)
Total length (Mb)	118.69	47.01	19.89	51.80
Average length per CNVR (Kb)	16.42	32.42	3.67	142.31
< 10 Kb	6228 (86.14%)	970	5184	74
≥ 10 Kb to < 50 Kb	626 (8.66%)	303	205	118
≥ 50 Kb to < 100 Kb	132 (1.83%)	59	21	52
≥ 100 Kb to < 500 Kb	199 (2.75%)	100	6	93
≥ 500 Kb to < 1 Mb	38 (0.53%)	18	0	20
≥ 1 Mb	7 (0.10%)	0	0	7

### Enrichment analysis of identified CNVRs

Enrichment analysis was used to analyze the function of CNVR-harboring genes further. There were 42, 58, and 34 significant GO terms (P < 0.05) related to hypoxia adaptation for BD, OL, and ZK, respectively. A total of 19 GO terms in the CNVRs were significantly enriched in at least two breeds, including two biological processes, 13 cellular components, and four molecular functions. These genes were mainly concentrated in the cellular components (Supplementary Table S5). These GO terms involved transcription corepressor activity (GO:0003714), NAD binding (GO:0051287), cytoskeleton (GO:0005856), polymeric cytoskeletal fiber (GO:0099513), antigen processing and presentation (GO:0019882), and cargo receptor activity (GO:0038024). The specific genes containing CNVR-harboring genes were analyzed for functional enrichment. The CNVR-harboring genes were mainly involved in sensory perception systems (GO:0004984), response to stimulus (GO:0050896), signal transduction (GO:0038023, GO:0007165, GO:0023052), and molecular structure (GO:0005622, GO:0044424, GO:0043229). Accordingly, another function of CNVR-harboring genes was analyzed. There were 36, 87, and 36 significant GO terms (P < 0.05) related to coat color for BD, GY, and OL, respectively. Only three GO terms in the CNVRs were significantly enriched in at least two breeds, including three biological processes (Supplementary Table S6). These GO terms involved hormone transport (GO:0009914), hormone secretion (GO:0046879), and regulation of hormone secretion (GO:0046883). The results showed that the CNVR-harboring specific genes were mainly involved in signal transduction (GO:0007165, GO:0023052), response to stimulus (GO:0051716, GO:0050896), and regulating process (GO:0050794, GO:0050789, GO:0065007).

**Figure 5:**
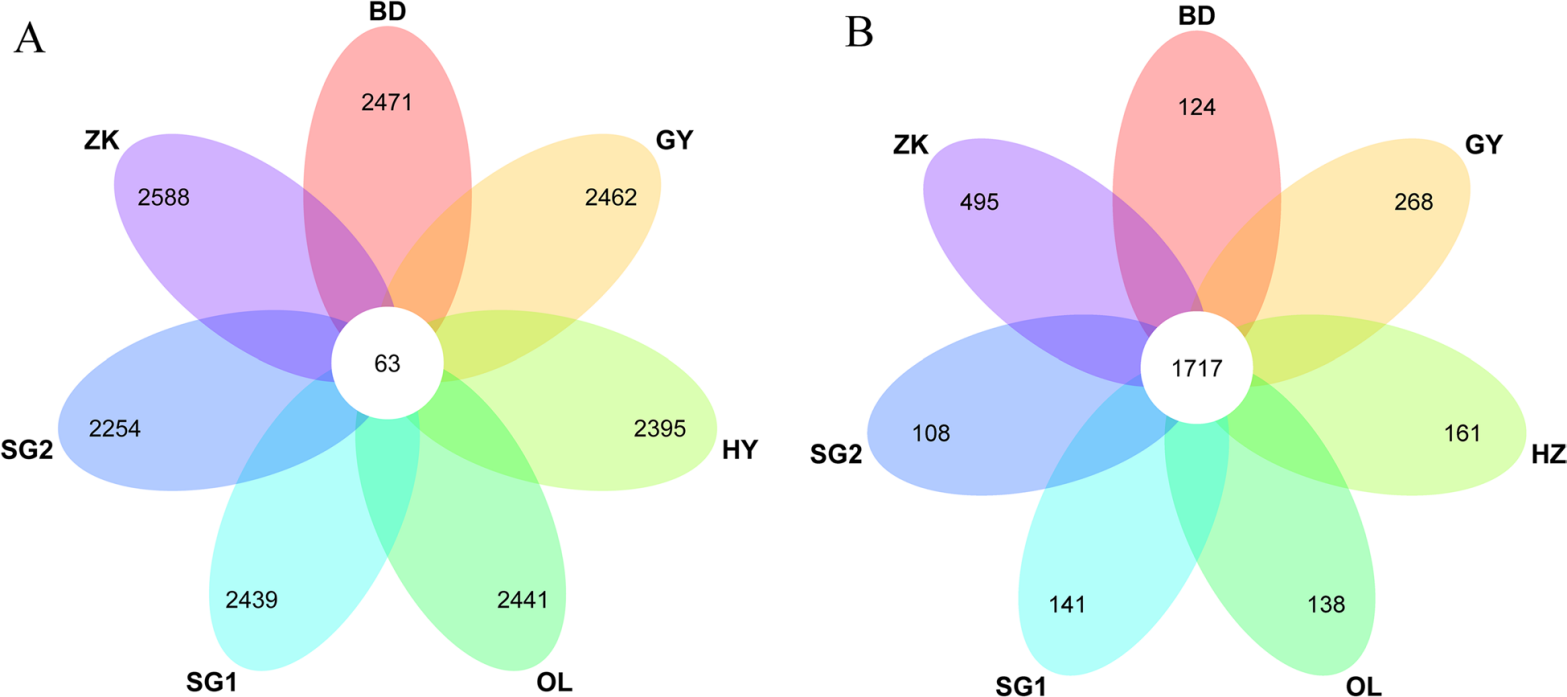
Venn diagrams of common selected regions (**A**) and corresponding genes (**B**) among different comparisons.

The number of shared CNVRs and genes among sheep breeds are shown in a Venn diagram. There were 2588, 2254, 2439, 2441, 2395, 2462, and 2471 group-specific CNVRs associated with 495, 108, 141, 138, 161, 268, and 124 genes in ZK, SG2, SG1, OL, HZ, GY, and BD, respectively, while 63 identical CNVRs (1717 co-selection genes) were shared among sheep breeds (Fig. 5A and B, Supplementary Table S4). Furthermore, the higher shared CNVRs of GY with SG, HZ, and ZK were consistent with the distribution of these sheep breeds. GY had the largest number in different groups of Tibetan sheep in Qinghai Province, accounting for approximately 90%. The breed has been raised in diverse geographical regions and exhibits adaptability to various environmental conditions. Moreover, the occurrence of gene flow among breeds and shared ancestral components may contribute to an augmentation in common copy number variant regions (CNVRs).

### Genetic stratification of CNVRs population

**Figure 6:**
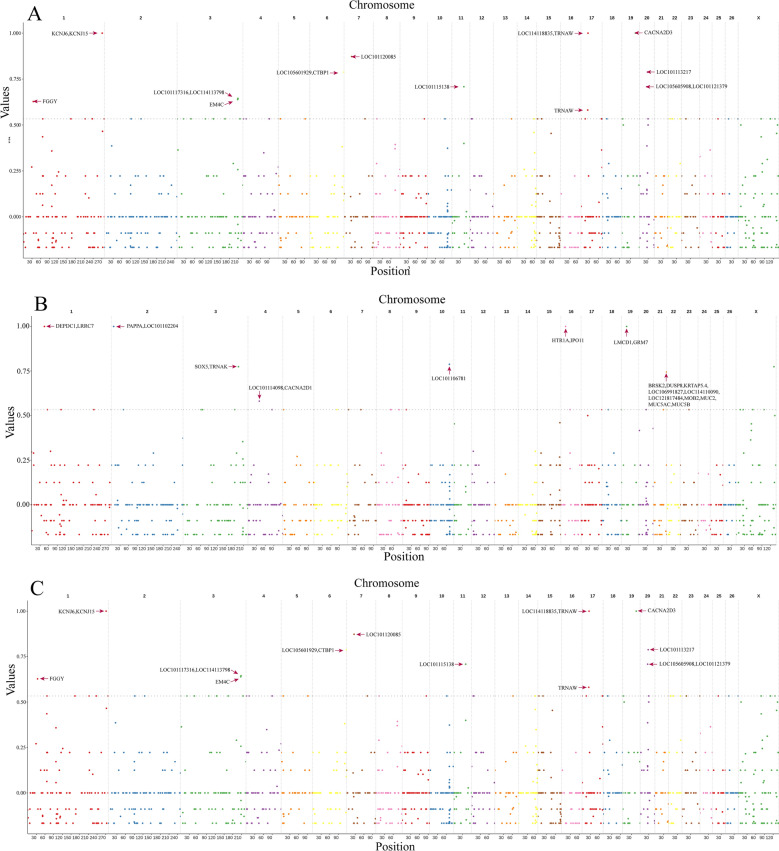
Genome wide Vst value plots for CNVRs. The horizontal gray dashed line represent top 1% of VST value.

To identify the loci of different Tibetan sheep breeds under adaptive selection, selective sweep analysis based on genotyped copy number for all CNVRs were performed using the Vst value. We selected the Vst value of 1% CNVRs as the candidate CNVRs. As a result, the high-altitude and low altitude Tibetan sheep showed extreme stratification in many of their chromosomes (Vst ≥ 0.53) (Fig. 6A, Supplement Table S7), with the most significant loci on chromosomes 6 and 19, in the *CACNA2D3*, *LOC105601929,* and *CTBP1* gene. These stratified loci had two CNVs (6: 117834601–117846100 and 19: 46784401–46785900), which were “duplication” and “deletion” types. Further, the KEGG functional enrichment analysis revealed that the genes annotated in CNVRs were predominantly enriched in the Oxytocin signaling pathway, Notch signaling pathway, and Cardiac muscle contraction signaling pathway (P < 0.05) (Supplement Table S8). Similarly, we identified stratified loci for coat color (Vst ≥ 0.53) (Fig. 6B, Supplement Table S9) and body weight (Fig. 6C, Vst ≥ 0.70) (Supplement Table S10), including a significant variation on chromosomes 16 and 23, in the *HTR1A*, *TRNAS-GGA*, and *PIK3C3* gene. These loci contain three CNVs (16: 16008501–16010200, 9: 10536701–10543500, 23: 14978401–14981600), which were all “deletion” types. KEGG analysis results indicated that these CNVRs were enriched in the cAMP signaling pathway and the Autophagy signaling pathway, respectively (P < 0.05) (Supplement Table S11 and S12).

### qPCR validation of CNVRs

**Figure 7:**
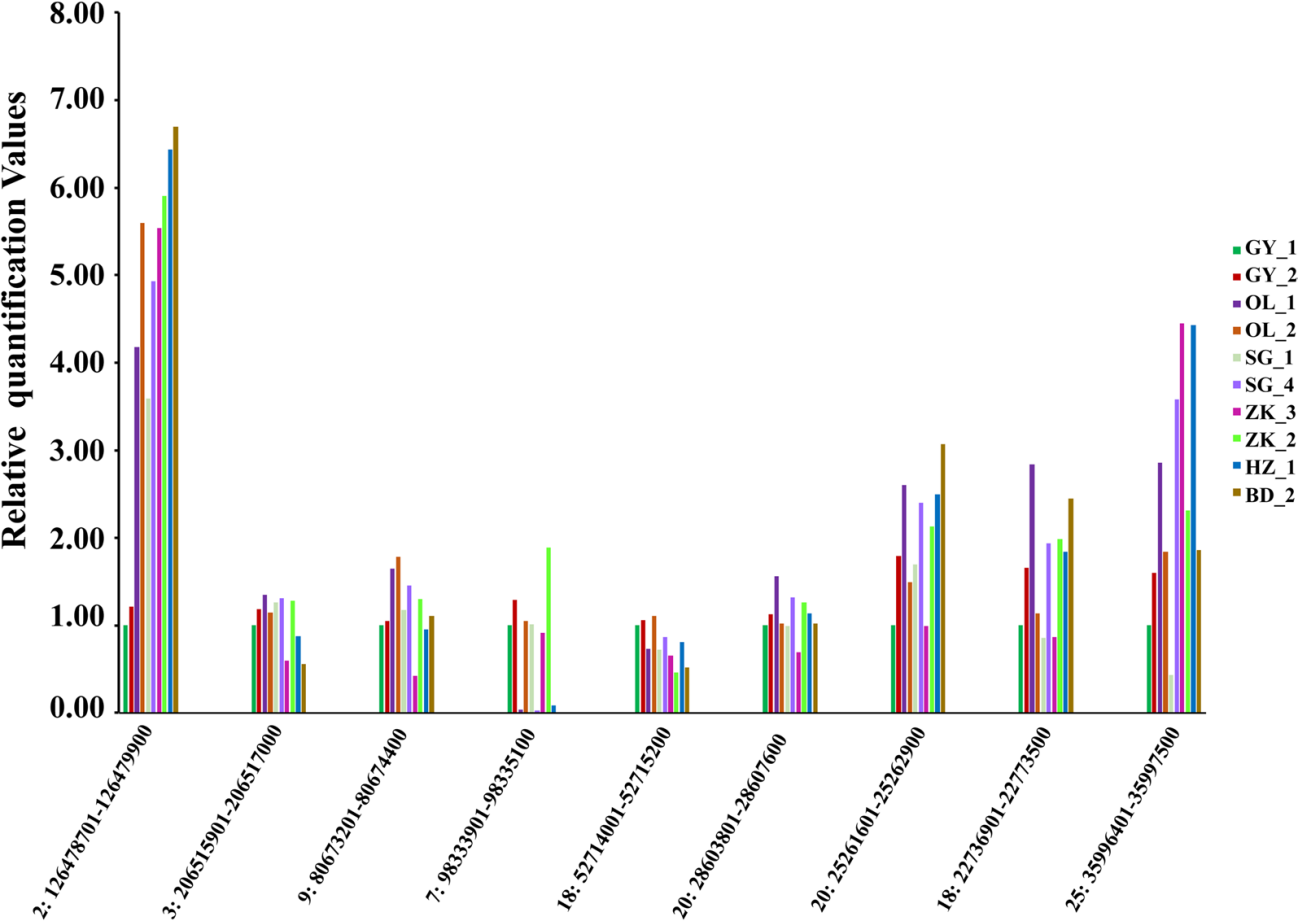
qPCR validation of selected CNVRs. The y-axis shows the Relative quantification Values obtained by qPCR, while the x-axis indicates the sample names in the different CNV regions.

We randomly selected nine CNVRS and verified the accuracy of our CNVR prediction by qPCR in 10 sheep samples. These randomly selected CNVRs were confirmed by the predictions made by PennCNV. The verification results are shown in Fig. 7.

## Discussion

The ubiquity of copy number variation in mammalian genomes has been recognized. Many CNVs cause differences in gene expression levels, so that CNVs may account for a large proportion of normal phenotypic variation^4^, possibly through dose regulation, number of copies, and location effect regulation^15,16^. The creation of new genes in regions with variable copy numbers may diversify the repertoire of these genes in response to rapidly changing environments^17^. Studies of CNVs in ruminants such as cattle and sheep show that phenotypes, reproductive and economic traits such as hypoxic adaptation, litter size, meat quality, disease resistance, and body weight are strongly correlated with CNVs^18–22^. We used whole genome sequencing to identify CNVRs events in different indigenous Tibetan sheep groups for the first time. We note that several genes of unique CNVRs in hypoxic-adapted Tibetan sheep are involved in regulating the Oxytocin signaling pathway, Notch signaling pathway, and Cardiac muscle contraction signaling pathway under different environmental selection pressures. There are also CNVRS-specific genes for coat color and reproductive traits. These genes are extensively involved in phenotype formation and environmental adaptation of Tibetan sheep. The phenotypic differentiation and environmental adaptation of Tibetan sheep from diverse geographical regions underscore the significance of genetic determinants in shaping phenotypic specificity through targeted regulation of distinct genes.

We also found that many CNVRs genes in Tibetan sheep were significantly enriched in the sensory perceptual system, response to stimulus, and signal transduction GO cluster, which are responses of animals to hypoxic environments. Tibetan sheep are mainly grazed, and their sensory perceptual system is significantly enriched, which brings potential advantages in adapting to the shortage of forage grass and avoiding the attack of predators^23,24^. In addition, the investigation into the gene content of the CNV region in yaks revealed a significant expansion of the gene family associated with sensory perception, which played a pivotal role in adapting to rapid environmental changes^25^. Response to stimulus plays an important role in plateau adaptation as a regulatory factor to cope with a hypoxic environment^26^. Signal transduction can transmit extracellular signals into the nucleus, including genes related to proliferation, differentiation, and apoptosis. Therefore, yak populations under different ecological conditions at different altitudes can undergo natural selection in the signal transduction systems and exhibit strong adaptability to hypoxia^27^.

The gene *CACNA2D3*, which is functionally associated with blood circulation and cardiac contraction, exhibits the potential to mitigate blood flow resistance and reduce the risk of cardiac events in high-altitude populations, making it a promising candidate for high-altitude adaptation^28^. The experimental evidence suggests that PKD2's functional partners, namely *CACNA1C*, *CACNA2D1*, and *CACNA2D3*, have the ability to interact with other calcium channels in order to form functional complexes. Conversely, a point mutation identified at the PKD1l1 site rks disrupts its interaction with *PKD2*. This particular mutation has been found to result in right lung isomerism, cardiac outflow defects, significant edema, and a mortality rate of 15.5%, these effects can impact calcium circulation and have detrimental consequences on cardiac development and function^29,30^. Other studies focused on the combined analysis of microRNA (miRNA) and *CACNA2D3*, indicating that the target of miR-27a is the ER-located Ca^2+^ transporter *CACNA2D3*, miR-27a may inhibit the expression of *CACNA2D3* by directly and sequence-specific binding to the 3 '-UTR of *CACNA2D3*, thereby inhibiting Ca^2+^ mediation^31^. Furthermore, *CTBP1* is involved in glycolytic metabolism. Hypoxia-induced upregulation of CTBP enhances glycolysis, leading to increased expression of hypoxia-inducible factor (HIF) family target genes. This alteration in the cellular microenvironment promotes cell growth under conditions of hypoxic adaptation and suggests that mutations in this gene may be preferentially selected to optimize glycogen utilization as an energy source during hypoxia and anaerobic glycolysis^32,33^. In our study, the *CACNA2D3* and *CTBP1* gene was enriched in the Cardiac muscle contraction, Hypertrophic cardiomyopathy, and Notch signaling pathway. After long-term adaptation to the high-altitude environment, the oxygen-carrying capacity of blood is increased through the blood circulation and signal transduction system, and the remarkable ability to adapt to the low-oxygen environment is gradually acquired^34,35^. The aforementioned genes confer advantages in response to the selective pressure of hypoxic adaptation in high-altitude Tibetan sheep.

Serotonin (5-hydroxytryptamine; 5-HT) is an important mediator in the interaction between epidermal melanocytes and the neuroendocrine system^36^. There is strong evidence in mice that the serotoninergic system promotes hair growth and the development of pigmentation through the 5-HT1A/1B receptor, which is mediated by the 5-HT1A/1B receptor, and confirms the expression of 5-HTR1A in skin melanocytes^37^. This result is consistent with detecting of 5-HT1A on mammalian melanocytes as a signaling substance mediating neurocutaneous interactions at both organ and cellular levels^36^. We found that *PIK3C3* and *TRNAS-GGA* genes are associated with fat deposition and body weight in the unique CNVRs of BD Tibetan sheep. The average daily gain, backfat thickness, and intramuscular fat content of the fifth generation of Duroc pigs exhibited significant improvements compared to those of the first and second generations due to enhanced breeding programs, indicating a potential correlation between changes in the frequency of *LEPR* and *PIK3C3* alleles in this population and selective breeding practices. Association analysis conducted on individual candidate genes revealed that *PIK3C3* exerted pleiotropic effects on growth and fat deposition, with a positive additive effect observed specifically on average daily gain (multi-candidate gene effect evaluation)^38,39^. The *TRNAS-GGA* gene is located on chromosome 6 and is considered a potential candidate gene associated with birth weight. The protein encoded by the *TRNAS-GGA* gene plays crucial roles in regulating cell cycle progression, DNA replication, cell proliferation and apoptosis, as well as lipid and carbohydrate metabolism^40,41^. In additive and dominant models, it was found that polymorphisms in the flanking region of the *TRNAS-GGA* gene were correlated with carcass weight^42^. BD sheep habitat in the Qinghai-Tibet Plateau is above 3700 m, and BD belongs to the short-haired meat breed, which can only store fat to resist cold. The candidate genes under consideration have the potential to exert an influence on the phenotypic characteristics and production traits of sheep. However, further investigations are required to ascertain the precise mechanism through which these genes impact phenotypes.

## Conclusion

We have conducted genome-wide CNVS in different groups of local Tibetan sheep and found that the unique CNVRs functional genes related to phenotypic traits and important economic traits were strongly selected. From this, we identified some candidate genes. The data obtained in this study will establish a solid foundation for future breeding programs aimed at enhancing desirable traits and conserving the genetic diversity of Tibetan sheep. Moreover, it will provide robust support for identifying candidate genes associated with economically important traits, thereby expediting the breeding process and augmenting the effectiveness of genetic improvement.

## Materials and methods

### Animal and sample collection

We sampled ear tissues from 28 female sheep of six geographical and phenotypic representative Tibetan sheep breeds (2-year-old ewes) from different geographical regions in Qinghai Province, including grassland sheep (GY), black sheep (HZ), Qumaari speckled (BD), Zeku sheep (ZK), Oula sheep (OL),valley sheep (four polled (SG1) and four horned (SG2)), sample information, such as species names, codes, sampling sites and altitude, longitude and latitude, and phenotype/feature, is shown (Fig. 1, Supplement Table S1). All tissue samples were preserved in 95% alcohol and stored at − 80 °C for later genomic analysis.

### Whole-genome sequencing and quality control

Total genomic DNA was extracted from samples, and at least three µg genomic DNA was used to construct paired-end libraries of 2 × 150 bp using paired-end sequencing. These libraries were sequenced using the Illumina NovaSeq6000 at Personalbio (Shanghai, China). FASTP^43^ Toolkit v0.18.0 was used for quality control of the raw reads according to three stringent filtering standards: (1) removing reads with ≥ 10% unidentified nucleotides (N); (2) removing reads with > 50% bases having Phred quality scores of ≤ 20; and (3) removing reads aligned to the barcode adapter.

### Read mapping

The Burrows–Wheeler Aligner (BWA) was used to align the clean reads from each sample against the reference genome (https://www.ncbi.nlm.nih.gov/genome/?term=Ovis%20aries) with the settings ‘mem 4 − k 32 − M’, where − k is the minimum seed length and − M is an option used to mark shorter split alignment hits as secondary alignments^44^. SAMtools and PICARD were used for subsequent processing to remove duplicates^45^.

### CNV calling, CNVR determination and annotation

CNVnator software (version 0.3.3) was employed to detect potential deletions and duplications in the whole genome, and a bin size of 100 bp was selected. The CNVs were tested according to Abyzov's recommended standards^46^. Then, the quality of the filter was applied to the CNVs with p value < 0.01 (P value calculated using t-test statistics), sizes > 1 kb, and fractions of mapped reads with zero quality (q0) < 0.5. The CNV was combined from different individuals into CNVS, as described by Redon et al., (2006) who classified them as “deleted” (CN < 0.4), “conserved” (0.4 ≤ CN ≤ 1.6), or “duplicated” (CN > 1.6)^47^. Gene annotation was performed for the CNVRs, and the CNV loci were annotated using ANNOVAR software.

### Enrichment analyses

Kyoto Encyclopedia of Genes and Genomes (KEGG) and Gene Ontology (GO) were analyzed in the DAVID database^48^, and P value ≤ 0.05 was considered significant enrichment of candidate genes.

### qPCR validation of CNVRs

Nine CNVRS were randomly selected to verify copy number polymorphisms by quantitative PCR (qPCR) in 10 sheep. The primers used in qPCR were designed by Primer 5.0 for those fragments (Supplementary Table S13). The 2^−ΔΔCT^ method was used to calculate the copy number of the target gene^49^ and actin was used as an internal reference gene to normalize the data. We constructed the standard curve of each CNVR, the experimental procedures were performed according to the manufacturer’s instructions, and CNVs were verified in the 2 × SYBR real-time PCR system (Roche). The Real-time PCR reaction was completed in a 20 µL system with the following procedure: 10 µL 2 × SYBR Real-Time PCR premixture, 0.4 µL forward primers, and 0.4 µL reverse primers, and CDNA 1 µL, RNAse free dH_2_O up to 20 µL. The PCR conditions were as follows: first step 95 °C for 5 min, followed by 40 cycles at 95 °C for 15 s and 60 °C for 30 s. Ten biological replicates were performed for each CNVR segment.

### Ethic approval

This study followed the recommendations of the “Regulations for the Management of Affairs Concerning Experimental Animals” (Ministry of Science and Technology, China, revised in June 2004). The study was approved by the Animal Care and Use Committees of the Northwest Institute of Plateau Biology, Chinese Academy of Sciences. The animals are not harmed during sample collection. The study was conducted in accordance with the ARRIVE Guidelines.

### Supplementary Information

Supplementary Table S1.

Supplementary Table S2.

Supplementary Table S3.

Supplementary Table S4.

Supplementary Table S5.

Supplementary Table S6.

Supplementary Table S7.

Supplementary Table S8.

Supplementary Table S9.

Supplementary Table S10.

Supplementary Table S11.

Supplementary Table S12.

Supplementary Table S13.

## Data Availability

The raw reads produced in this study were deposited in the NCBI SRA with the accession number SRA SUB10967785 under Bio-project PRJNA1015299 and PRJNA1019076.
